# Apparent premature battery depletion in a third-generation subcutaneous implantable cardioverter-defibrillator caused by charge count–based longevity estimation failure: A previously unreported mechanism

**DOI:** 10.1016/j.hrcr.2026.02.023

**Published:** 2026-03-09

**Authors:** Fawzi Kerkouri, Vincent Mansourati, Hugo Hager, Pierre-Yves Quedinel, Valérie Valls-Bertault, Jacques Mansourati

**Affiliations:** 1 Department of Cardiology, University Hospital of Brest, Brest, France; 2 Univ Brest, Laboratoire ORPHY EA 4324, Brest, France

**Keywords:** Subcutaneous implantable cardioverter-defibrillator, S-ICD, Cardiac implantable electronic devices, Early battery depletion, Longevity, Device malfunction, Remote monitoring, Postmarket surveillance, Ventricular arrhythmia, Sudden cardiac death

**Key Teaching Points**

• Novel mechanism of subcutaneous implantable cardioverter-defibrillator (S-ICD) premature battery depletion: “Virtual” charge counting can mislead the battery algorithm, causing a falsely fixed low percentage.
• Diagnostic uncertainty: Battery voltage/charge duration cannot be assessed during this phase, creating doubt about battery integrity and creates a replace-vs-surveil dilemma.
• Surveillance needed: Systematic remote monitoring is essential pending larger evaluation of third-generation S-ICDs’ battery performance.

## Introduction

Subcutaneous implantable cardioverter-defibrillator (S-ICD) generator longevity typically ranges from 5 to 7 years, substantially shorter than the 10–15 years observed with contemporary transvenous implantable cardioverter-defibrillators (ICDs).^1^ Premature battery depletion (PBD) has emerged as an important device-related issue in first- and second-generation S-ICDs, with incidences of 3%–10% at 5 years.^1^, ^2^, ^3^, ^4^ This phenomenon has been far less frequently described with newer third-generation devices incorporating improved technology, with an estimated incidence of ∼2% by 3 years.^5^^,^^6^ Known mechanisms include true battery failure owing to hydrogen-induced low-voltage capacitor compromise and false indicators from incorrect timestamp-related display errors.^7^^,^^8^ We report what seems to be the first instance of a previously unrecognized mechanism: apparent PBD caused by erroneous counting of nonexistent charge cycles.

## Case report

A 73-year-old man with dilated cardiomyopathy (left ventricular ejection fraction 30%), paroxysmal atrial fibrillation, chronic kidney disease (estimated glomerular filtration rate 25–32 mL/min), type 2 diabetes mellitus, hypertension, hypothyroidism, and obstructive sleep apnea underwent S-ICD implantation in 2017 for primary prevention. The generator was replaced on April 24, 2025, at normal end of life with a third-generation Emblem A219 device (serial number 324597) with a successful defibrillation testing at 60 J (impedance at 68 Ω), without any shock delivered since the initial implant. The patient was not enrolled in remote monitoring.

**Figure 1:**
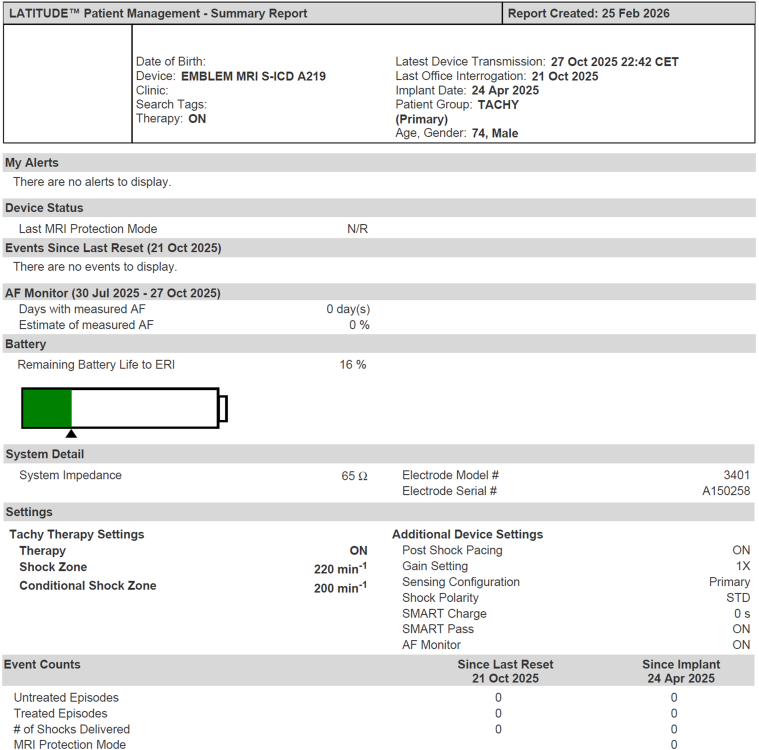
Device interrogation 5 months after third-generation Emblem A219 implantation showing a remaining battery capacity of 16%. AF = atrial fibrillation; ERI = elective replacement indicator; MRI = magnetic resonance imaging.

During a hospital admission for acute heart failure, routine device interrogation on October 21, 2025—only 5 months after generator replacement—showed a remaining battery capacity of 16%, with no shocks delivered, good signal quality, no oversensing or noise, normal lead impedance, and no evidence of repetitive capacitor charging. This represented a loss of ∼84% capacity within 5 months (Figure 1), suggesting severe early depletion. The device was not under advisory. The patient had not undergone magnetic resonance imaging or therapeutic radiotherapy, nuclear medicine treatment, or any known occupational exposure to ionizing radiation nor exposure to electromagnetic interference. The event was reported to materiovigilance authorities and the manufacturer.

Boston Scientific, through its central technical team in the United States, adjudicated the case and explained that the A219 uses a 2-phase algorithm to estimate battery longevity: for approximately the first two-thirds of its expected service life, the estimate is based on time since implant and the number of capacitor charges; in the final third, it switches to a voltage- and charge time–based estimation. In this case, manufacturer analysis revealed a software error leading to multiple “virtual” charge cycles being counted, although no corresponding therapies had actually been delivered. These internal counters are not accessible during routine interrogation and were identified only through dedicated manufacturer-level analysis.

Regarding battery performance, the manufacturer’s conclusion was based primarily on the nature of this error in the longevity algorithm. Because no real capacitor charges had occurred and there was no clinical or device-based evidence of increased energy use, they inferred that there was no reason for true battery depletion and therefore considered the battery cell itself to be intact. When we specifically requested confirmation of battery integrity through direct assessment of battery voltage or internal resistance, we were informed that these metrics could not be retrieved from the device during this phase of the algorithm. However, the manufacturer anticipated that, once the cell genuinely reached the last third of its life and the algorithm switched to the voltage-based mode, the displayed percentage would again become accurate. In the meantime, the device would continue to display a fixed battery status of 16% without reliable correction. Importantly, even if shocks were to be delivered during this initial phase, the displayed 16% value would remain unchanged because further charge cycles would not be counted. To their knowledge and ours, this was the first reported instance of this behavior.

**Figure 2:**
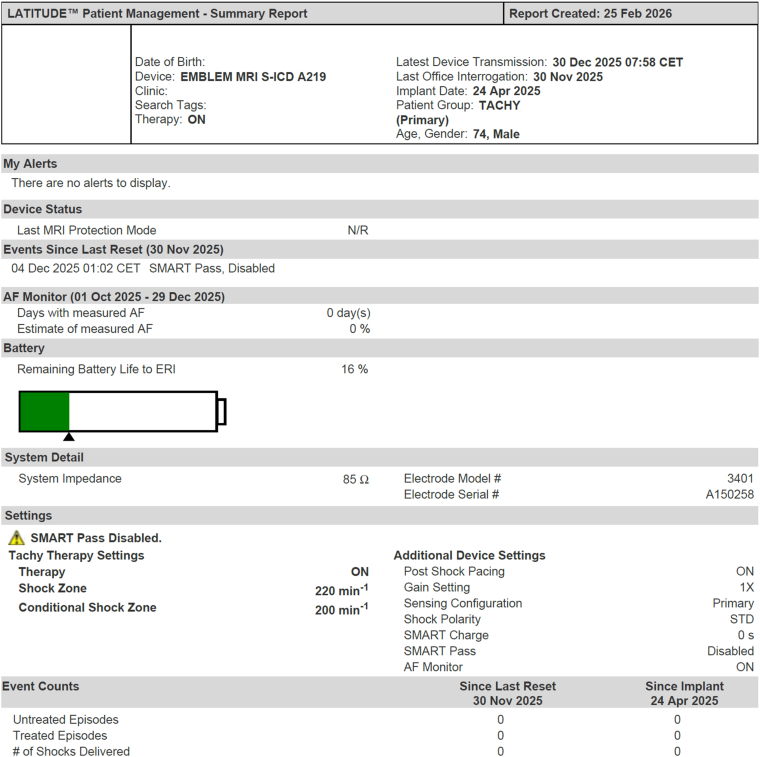
Device interrogation 7 months after third-generation Emblem A219 implantation fixed displayed battery capacity of 16%. Abbreviations same as Figure 1.

**Figure 3:**
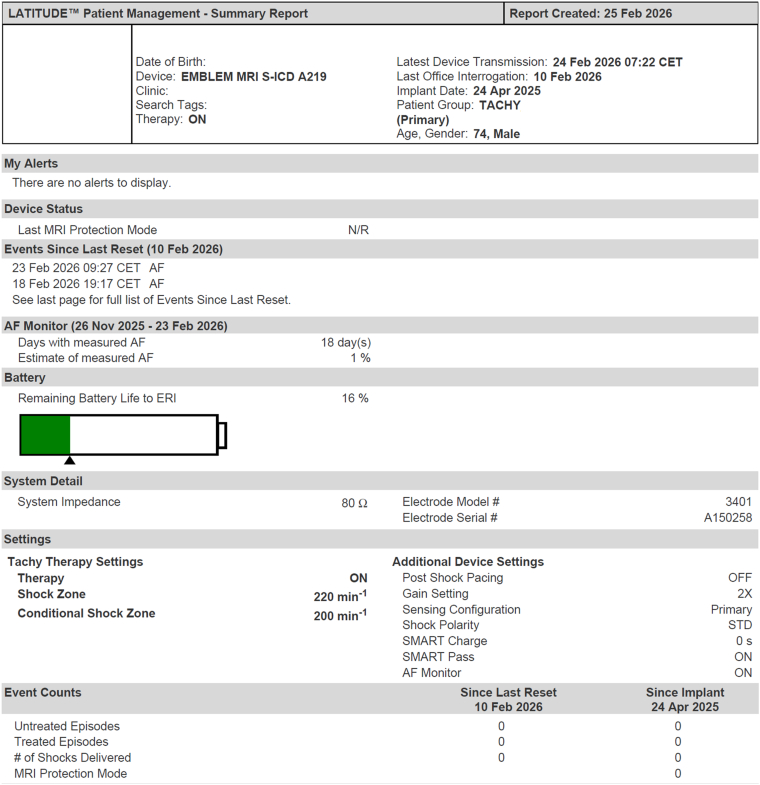
Device interrogation 9 months after third-generation Emblem A219 implantation fixed displayed battery capacity of 16%. Abbreviations same as Figure 1.

After this finding, the device was enrolled in remote monitoring via LATITUDE™ platform, with monthly remote transmissions and in-clinic checks scheduled every 12 months. Subsequent follow-up has shown no system alerts, and the displayed battery percentage has remained fixed at 16% (Figures 2 and 3).

## Discussion

To the best of our knowledge, this represents the first reported case of apparent PBD, occurring as early as 5 months after generator replacement with a third-generation Emblem A219 device. This finding reinforces the importance of remote monitoring for longevity assessment, even in nonadvisory S-ICDs.

The S-ICD represents a major step in ICD evolution, providing similar efficacy to transvenous ICDs for sudden cardiac death prevention while eliminating transvenous lead–related complications.^9^^,^^10^ However, inappropriate shocks remain slightly more frequent, and generator longevity is clearly shorter. PBD are not uncommon, with rates between 3% and 10% at 5 years depending on the cohort and definition used. Recent multicenter analyses have demonstrated a higher-than-expected incidence of PBD in S-ICD carriers.^1^ In devices produced before 2018, the mechanism of PBD is well characterized: low-voltage capacitor degradation leads to hydrogen release; hydrogen accumulation subsequently compromises capacitor function and accelerates battery drain.^6^ Large registries estimate PBD at approximately 10%–15% at 5 years in devices under advisory and up to 29% when using a clinical definition of rapid drop in longevity.^2^, ^3^, ^4^ From August 2018 onward, the low-voltage capacitor was redesigned and PBD has rarely been observed in these later series.^6^ Nevertheless, to the best of our knowledge, no case of PBD has been reported within the first year after implantation or generator replacement, and no failure of the electronic battery estimation system has previously been described.

In our case, the device was a postadvisory model and seemed to show extremely rapid decline within 5 months—the earliest apparent failure reported. Technical analysis by the manufacturer did not confirm capacitor failure. The patient had no shocks, no inappropriate sensing, normal lead impedance, and no evidence of frequent capacitor charging; thus, physiological or usage-related causes of depletion were unlikely. The absence of magnetic resonance imaging exposure or significant electromagnetic interference further argued against external factors. Instead, manufacturer identified a previously undescribed mechanism: a battery estimation algorithm failure owing to erroneous counting of nonexistent (“virtual”) charge cycles during the initial, time-/charge-based phase of the longevity algorithm. In this phase, the displayed percentage was inaccurate, whereas true battery capacity was considered normal and expected to be corrected only after the device reached the later, voltage-based phase 3–4 years after implant. Therefore, the manufacturer did not recommend generator replacement and advised simple monitoring.

However, this situation is clinically problematic. First, the displayed battery percentage remains fixed at 16%; even if real shocks are delivered before transition to the voltage-based phase, these will not be registered in the counter and the display will not change, raising concern that the device could approach true depletion while still indicating 16%. Second, the inability to directly verify battery voltage during this first phase makes it difficult to fully exclude a true battery problem and undermines confidence in long-term protection. Together, these uncertainties create a management dilemma between prophylactic generator replacement “by prudence” and continued surveillance with an unreliable battery indicator. Given the absence of appropriate shocks for more than 8 years and the stable nature of the patient’s cardiomyopathy, we initially elected to pursue active surveillance rather than immediate prophylactic generator replacement, with re-evaluation over roughly 12 months. Should the clinical situation change—such as the occurrence of ventricular arrhythmias or progression of heart failure—our strategy would be reassessed and generator replacement reconsidered.

## Conclusion

This case describes the first reported example of apparent PBD in a third-generation Emblem A219 S-ICD, ultimately attributed to a previously unrecognized failure of the battery longevity estimation algorithm rather than true battery failure. This situation creates a practical management dilemma, given that clinicians must choose between prophylactic generator replacement and continued surveillance despite a persistently misleading battery indicator. These findings highlight the need for transparent battery estimation algorithms, systematic remote monitoring, and dedicated postmarket evaluation of third-generation S-ICDs to ensure reliable long-term protection.

## Disclosures

Jacques Mansourati has received research fees and advisory board honoraria from all ICD manufacturers. Vincent Mansourati and Hugo Hager have received fees from ICD manufacturers. These fees were not related to this study. All other authors report no conflicts of interest.
